# Tibial plateau fractures: compared outcomes between ARIF and ORIF

**DOI:** 10.1007/s11751-012-0148-1

**Published:** 2012-10-20

**Authors:** C. Dall’Oca, T. Maluta, F. Lavini, M. Bondi, G. M. Micheloni, P. Bartolozzi

**Affiliations:** Department of Surgery, Orthopaedic and Traumatology Clinic, G.B. Rossi Hospital, University of Verona, Piazzale Scuro 10, 37134 Verona, Italy

**Keywords:** Tibial plateau fractures, ARIF, Arthroscopically assisted reduction, Post-traumatic arthrosis, External fixation

## Abstract

The purpose of this study is to compare arthroscopic assisted reduction internal fixation (ARIF) treatment with open reduction internal fixation (ORIF) treatment in patients with tibial plateau fractures. We studied 100 patients with tibial plateau fractures (54 men and 46 women) examined by X-rays and CT scans, divided into 2 groups. Group A with associated meniscus tear was treated by ARIF technique, while in group B ORIF technique was used. The follow-up period ranged from 12 to 116 months. The patients were evaluated both clinically and radiologically according to the Rasmussen and HSS (The Hospital for Special Surgery knee-rating) scores. In group A, the average Rasmussen clinical score is 27.62 ± 2.60 (range, 19–30), while in group B is 26.81 ± 2.65 (range, 21–30). HSS score in group A was 76.36 ± 14.19 (range, 38–91) as the average clinical result, while in group B was 73.12 ± 14.55 (range, 45–91). According to Rasmussen radiological results, the average score for group A was 16.56 ± 2.66 (range, 8–18), while in group B was 15.88 ± 2.71 (range, 10–18). Sixty-nine of 100 patients in our study had associated intra-articular lesions. We had 5 early complications and 36 late complications. The study suggests that there are no differences between ARIF and ORIF treatment in Schatzker type I fractures. ARIF technique may increase the clinical outcome in Schatzker type II–III–IV fractures. In Schatzker type V and VI fractures, ARIF and ORIF techniques have both poor medium- and long-term results but ARIF treatment, when indicated, is the best choice for the lower rate of infections.

## Introduction

Tibial plateau fractures are complex injuries of the articular and the metaphyseal segments. Surgery is challenging due to the fracture patterns and the associated complications. The displacement of the bony fragments and pattern of involvement of subchondral bone and cartilage characterize the severity of the lesion and treatment strategy. The associated soft-tissue damage, knee instability, meniscal lesions and possibility of compartment syndrome also influence treatment methods [1–11].

Open reduction and internal fixation (ORIF) with plates and screws is an established method of treatment for complex fractures (Schatzker types V–VI). ORIF strategy has undergone refinement with the advent of external fixators into the treatment plan and introduction of low profile plates and anatomic periarticular implants [12–14]. External circular fixation is used for complex fractures with soft-tissue damage because of the advantage of being minimally invasive and a potential to reduce deep infection [8, 15–23]. External fixators are also used as a temporary stabilization frames across the knee joint for pain relief, provisional reduction and soft-tissue control; CT scans are obtained for pre-operative planning [17, 24, 25]. Recently, we have observed a progressive modification of the treatment from ORIF to arthroscopic assisted reduction and internal fixation (ARIF) [14, 24, 26–28]. Some authors recommend ARIF for Schatzker type I, II, III, IV whilst a few have done the same for type V and VI fractures [6, 9, 14, 23, 28–31].

The aim of this retrospective study was to compare the results obtained by ARIF versus ORIF treatment.

## Materials and methods

The study relates to patients with tibial plateau fractures treated between March 2000 and December 2009. There were 100 patients, 54 men and 46 women with a mean age of 51 years (range 13–77), who underwent operative surgery. There were 14 cases of type I fracture, 12 type II, 44 type III, 8 type IV, 12 type V and 10 type VI, according to the Schatzker classification [32]. In order to decide treatment, all patients were assessed using X-rays and CT scans [25, 33]. If an associated meniscal tear was present, the patient was treated with the ARIF technique. Otherwise the ORIF technique was used, avoiding arthrotomy where possible. For Schatzker V and VI fractures, the ARIF technique was used only in selected cases where a low degree of comminution was present.

There were two groups: group A (ARIF; composed of 50 patients of whom 23 were males) and group B (ORIF; composed of 50 patients of whom 31 were males). The exclusion criteria were: open fractures; pathologic fractures; significant pre-existing degenerative joint disease; severe systemic illness (active cancer, chemotherapy, renal failure or other comorbidities that contraindicate surgery) or a neurological condition that would interfere with rehabilitation. The follow-up period ranged from 12 to 116 months, with a mean of 73, 27 months. No patients were lost to follow-up.

Sixty-four patients were injured in traffic accidents, 24 in sport injuries (ski, motorbike, bicycle and rugby) and 12 by a simple fall. Eighteen of them had associated fractures (2 clavicle fractures, 12 distal radial fractures and 4 proximal humerus fractures) all of which were treated with conservative methods. The patients were evaluated as follows: soft-tissue condition using the Tscherne classification, sensorimotor function of the limb by a clinical neurological examination and vascular status by Doppler.

The mean time between day of admission and surgery was 4 days (range 2–10 days) and the timing of surgery was influenced by the patient’s general and the soft-tissue envelope conditions, in particular significant oedema and skin blisters [34].

Type I fractures were treated using cannulated screws, type II by plates and screws, type III by cannulated screws or plates and screws. Type IV, V and VI were treated by plates and screws with cannulated screws added if needed; in three Schatzker V and three Schatzker VI fractures, double plates were used through two incisions; for the remainder of type V and VI fractures, we used a circular external fixator as definitive treatment as the metaphysis was highly comminuted or the soft-tissue conditions were of Tscherne grade 3 [35]. With high energy fractures, it was necessary to wait for better local conditions in order to perform internal synthesis and for this reason temporary bridging external fixators were used.

In this series, 6 cases (2 Schatzker V and 4 Schatzker VI fracture) were treated with circular fixators and 10 (5 Schatzker V and 5 Schatzker VI fracture) with temporary bridging external fixators to be followed by ORIF.

Joint distension during ARIF treatment was accomplished by intra-articular fluid infusion by gravity with a third portal used for venting to prevent extravasation increases in joint pressure.

Bone grafts taken from the iliac crest were used in 3 cases (3 %).

Knee motion was allowed 10 days after surgery in both groups. Partial weight bearing was permitted at an average of 6.3 weeks post-operatively and full weight bearing at 9.0 weeks in both groups (Table 1).

The patients were evaluated clinically and radiologically using the Rasmussen and HSS (The Hospital for Special Surgery knee-rating score) systems. This provided a record of functional and anatomic results after treatment [36, 37]. The follow-up protocol included analysis of subjective complaints and objective clinical findings. Radiographic evaluations were done pre-operatively, at 3, 6 months and 1 year post-operatively. Standing X-rays of the knee were evaluated at each year interval from surgery in order to detect joint depression, articular degeneration and axial changes.

**Table 1:** Patient’s data, treatment and associated lesions

Classification	Schatzker I (n.14)		Schatzker II (n.12)		Schatzker III (n.44)		Schatzker IV (n.8)		Schatzker V (n.12)		Schatzker VI (n.10)	
Treatment	ARIF	ORIF	ARIF	ORIF	ARIF	ORIF	ARIF	ORIF	ARIF	ORIF	ARIF	ORIF
Patient	4	10	7	5	26	18	5	3	4	8	4	6
Age	33.29	44.14	54.33	53.67	51.64	48.5	53.75	64.25	51.33	45	38.8	51.4
*Gender*												
M	2	6	4	3	10	13	3	2	3	3	1	4
F	2	4	3	2	16	5	2	1	1	5	3	2
*Side*												
R	1	7	2	1	14	14	3	1		6	4	3
L	3	3	5	4	12	4	2	2	4	2		3
*Treatment*												
Cannulated screws	4	10			14	7	1	2	3	5	3	4
Plate + screws			7	5	12	11	4	4	3	7	2	4
Circular external fixation									1	1	2	2
Transarticular external fixation									2	3	2	3
*Associated lesions*												
None		10		2	11	8						
Meniscus	4		5	2	13	10	3	2	4	7	4	3
ACL			2		2		2	1	1	2	2	2
PCL							1	1		1		
MCL				1					2		2	2
LCL								1				

## Results

In group A, the average Rasmussen clinical score is 27.62 ± 2.60 (range 19–30). Scores related to each Schatzker type of fractures are reported in Table 2. The following scores were obtained: 29.75, 27.71, 28.62, 26.4, 24.5 and 23.5, respectively, for Schatzker I, II, III, IV, V and VI types of fracture. In group B, the average Rasmussen clinical score is 26.81 ± 2.65 (range 21–30).

**Table 2:** Rasmussen, HSS scores and complications

Classification	Schatzker I (n.14)		Schatzker II (n.12)		Schatzker III (n.44)		Schatzker IV (n.8)		Schatzker V (n.12)		Schatzker VI (n.10)	
Treatment	ARIF	ORIF	ARIF	ORIF	ARIF	ORIF	ARIF	ORIF	ARIF	ORIF	ARIF	ORIF
Patient	4	10	7	5	26	18	5	3	4	8	4	6
*Rasmussen clinical assessment*												
Pain	5.75	5.7	5.42	5.2	5.69	5.44	5	4.33	4.75	4.5	4.5	4.67
Walking capacity	6	6	5.29	5.2	5.69	5.67	5.6	4.67	4.5	4.75	4.5	4.67
Extension	6	6	5.71	5.6	5.54	5.67	5.6	5.33	4.5	4.75	4.5	4.33
ROM	6	5.9	5.29	5.2	5.69	5.22	4.8	4.67	5	4.5	4.75	4.5
Stability	6	6	6	6	6	6	5.4	5.67	5.75	5.38	5.25	5.17
TOT	29.75	29.6	27.71	27.2	28.62	28	26.4	24.67	24.5	23.84	23.5	23.3
*Rasmussen radiological assessment*												
Depression	6	6	6	5.2	5.77	5.67	5.2	4	4	4.75	4	4.33
Condylar widening	6	6	5.43	5.6	5.77	5.89	6	5.33	5	5	4	4
Angulation (varus/valgus)	6	6	5.71	5.6	5.85	5.78	5.2	5.33	5	4	3.5	2.67
TOT	18	18	17.14	16.4	17.38	17.33	16.4	14.67	14	13.75	11.5	11
*HSS score*												
Pain	30	30	21.43	18	22.31	20.28	13	8.33	8.75	8.75	7.5	8.33
Function	12	11.8	11.14	11.6	11.54	11.67	11.2	10	10	10.5	9	8.67
ROM	17.5	17.4	16.57	16.8	17.23	16.44	15.2	15.33	15.25	13.75	14.5	14.5
Muscle strength	10	9.8	9.14	9.2	9.69	9.56	9.2	7.33	8.5	8.25	8	9
Flexion deformity	10	10	8.57	9.2	9.42	9.17	9	8.67	7.5	6.63	7.5	7.67
Instability	10	10	10	10	10	10	9	8.67	7.5	8.13	8.75	6.67
Subtraction	1	1	1	1	1.04	1	1	1	1.25	1.25	1.25	1
TOT	90.5	90	77.86	75.8	81.23	78.11	67.6	59.33	58.75	57.25	56.5	55.83
*Complications*												
Early complications												
SPE stupor				1								
TVP											1	1
Superficial infection										2		
Late complications												
Deep infection										1		1
Algodystrophy										2		2
Intolerance fixation			2	3	4	6	2	2	1	5	2	3

Analysing the clinical scores for each type of fracture, 29.6, 27.2, 28, 24.67, 23.84 and 23.3 were obtained, respectively, for Schatzker I, II, III, IV, V and VI types.

Using the HSS score, group A had 76.36 ± 14.19 (range 38–91) on average.

The HSS scores for each type of fracture were 90.5, 77.86, 81.23, 67.6, 58.75 and 56.5, respectively, for Schatzker I, II, III, IV, V and VI types.

In group B, the average HSS score was 73.12 ± 14.55 (range 45–91).

The scores for each type of fracture were 90, 75.8, 78.11, 59.33, 57.25 and 55.83, respectively, for Schatzker I, II, III, IV, V and VI types.

According to Rasmussen radiological results, the average score for group A is 16.56 ± 2.66 (range 8–18). The scores for each type of fracture were 18, 17.14, 17.38, 16.4, 14 and 11.5, respectively, for Schatzker I, II, III, IV, V and VI types. In group B, the average score was 15.88 ± 2.71 (range 10–18). The scores for each type of fracture were 18, 16.4, 17.33, 14.67, 13, 75 and 11, respectively, for Schatzker I, II, III, IV, V and VI types.

### Associated injuries and procedures

Sixty-nine of 100 patients in our study had associated intra-articular lesions. Of the remaining 31 patients, without associated lesions, 20 of them belonged to group B while 11 patients belonged to group A. A lesion of the meniscus was found in 57 knees: a medial meniscus tear in 13 knees; a lateral meniscus tear in 34 knees and bilateral meniscal tears in 10 knees. Thirty-two menisci were sutured, 21 partially resected and 4 totally removed.

Ruptures or avulsions of ligaments were found in 25 knees, including 14 anterior cruciate ligament avulsions, 3 posterior cruciate ligament ruptures, 1 lateral collateral ligament avulsion at the fibular insertion, 7 medial collateral ligament partial ruptures and 3 combination of anterior cruciate ligament and medial collateral ligament partial ruptures. Eight anterior cruciate ligament lesions were treated arthroscopically, 6 lesions were treated with a secondary reconstruction of the ligament. The lateral collateral ligament avulsion was fixed.

### Complications

There were no complications directly associated with arthroscopic procedures in group A. There were two cases of deep vein thrombosis: one in group A and one in group B. One patient who underwent ORIF treatment developed a common peroneal nerve neurapraxia which recovered fully in 4 months. There are no post-operative incidences of compartment syndrome in either group.

Two patients in group B had a superficial infection treated with antibiotic therapy after sample culture and identification of bacteria. Two deep infections occurred in ORIF group: one in a type V and one in a type VI fracture. The first (type V) was probably due to a proximal pin site infection from the temporary external fixator and was treated by removal of the device and substitution with an antibiotic-embedded cement spacer. The spacer was maintained for 8 months and, when there was no evidence of infection relapse through labelled-leucocyte scintigraphy and serological markers, a knee prosthesis was inserted. The second (Schatzker VI) healed in a cast with ongoing chronic infection despite fixation implant removal. Completion of treatment was not feasible in this case due to mental health issues and lack of compliance with the patient.

There were 10 cases of intolerance to the medial plates and 20 to the lateral plates: six patients in group B and four in group A needed the medial plates removed and 13 patients in group B and seven in group A needed the same for the lateral plates. No mechanical failures were observed. Four patients in group B developed algodystrophy which was treated with hyperbaric oxygen therapy and anti-osteoporotic drugs.

One case in group A (41 years old) had residual valgus angulation and arthritis after 1 year which was treated with uni-compartmental knee prosthesis. Two cases in group B (67 and 69 years old) developed degenerative arthritis with a significant post-traumatic valgus alignment and were treated with a total knee prosthesis (Table 2).

## Discussion

The standard of treatment for tibial plateau fractures is an anatomic reduction in the articular surface with stable fixation to allow early recovery of range of motion. It is also important to avoid ligamentous laxity in order to prevent late knee instability.

Several reports support arthroscopic management of tibial plateau fractures [4–6, 9, 11–13, 15–17, 19, 20, 22, 26, 28]. We observed that type I fractures had excellent results in both groups. There was no post-traumatic sequel from the approach. Schatzker II fractures, characterized by greater displacement and lateral cortex disruption, were associated often with a lateral meniscus lesion and an MCL or ACL lesion. Excellent results were obtained in both groups but with the ARIF technique, we were able to check and treat the associated injuries. We observed that patients treated by ARIF technique showed better values of ROM and sustained less pain than patients of group B, particularly within 12 months after surgery (Table 2). This was clinically important but was not statistically different due to the small numbers in each group. There was one early complication: a common peroneal nerve neurapraxia which recovered spontaneously after 4 months. Five cases (41.7 %) of late complications from the intolerance of the lateral implant were treated by plate removal.

In type III fractures treated by ARIF and ORIF, excellent results were obtained in both groups. There was a high incidence of lateral meniscus lesions, suggesting ARIF treatment being better suited in order to identify and treat these problems as well as aid in restoration of articular congruity. There were late complications: ten cases (22.7 %) of the lateral plates had to be removed. We noted that Schatzker III type fractures treated either by ARIF and ORIF techniques had better results than Schatzker II type fractures.

**Fig. 1:**
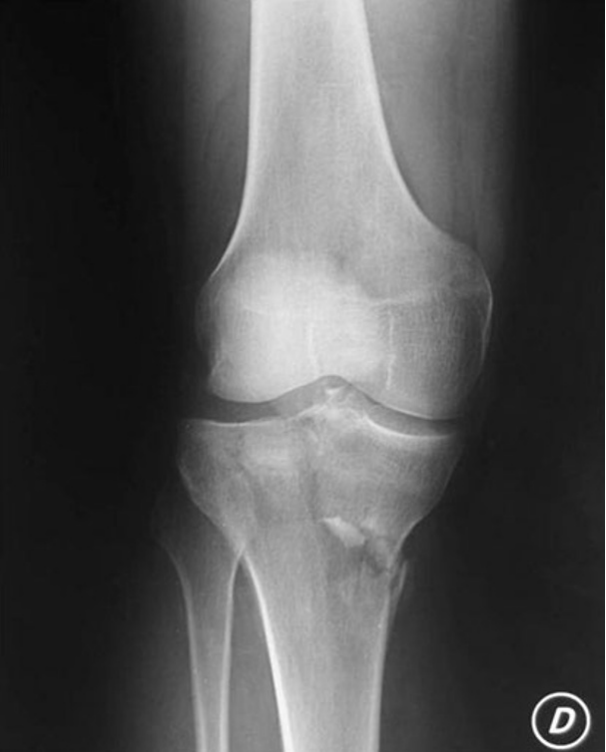
Schatzker type IV fracture, pre-op X-rays (AP)

**Fig. 2:**
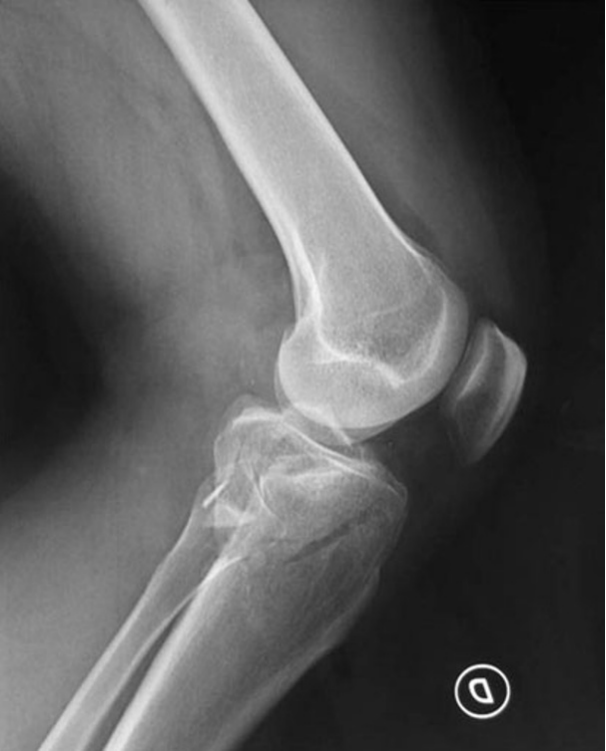
Schatzker type IV fracture, pre-op X-rays (LL)

**Fig. 3:**
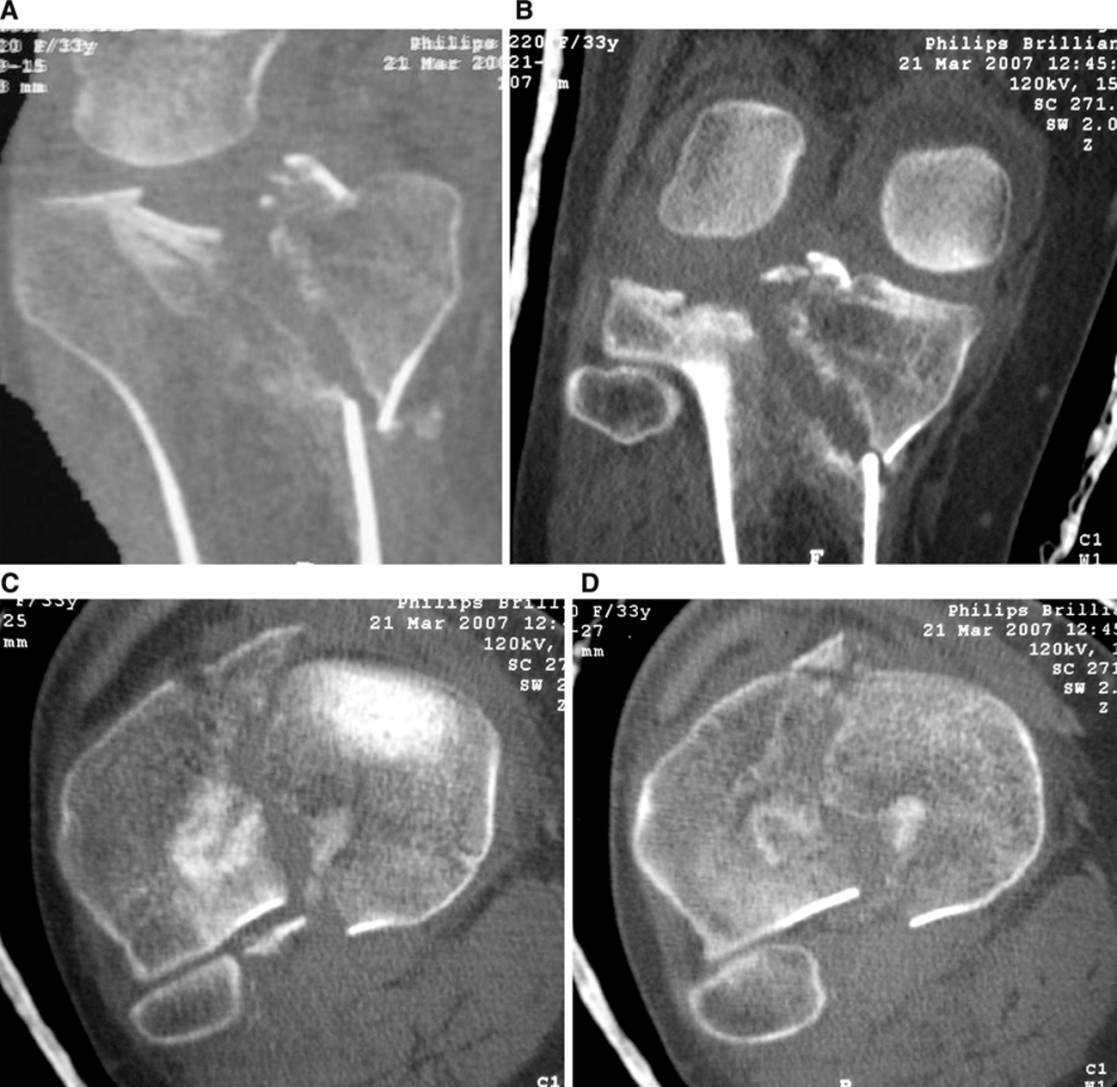
**a** Schatzker type IV fracture, CT scan. **b** Schatzker type IV fracture, CT scan. **c** Schatzker type IV fracture, CT scan. **d** Schatzker type IV fracture, CT scan

**Fig. 4:**
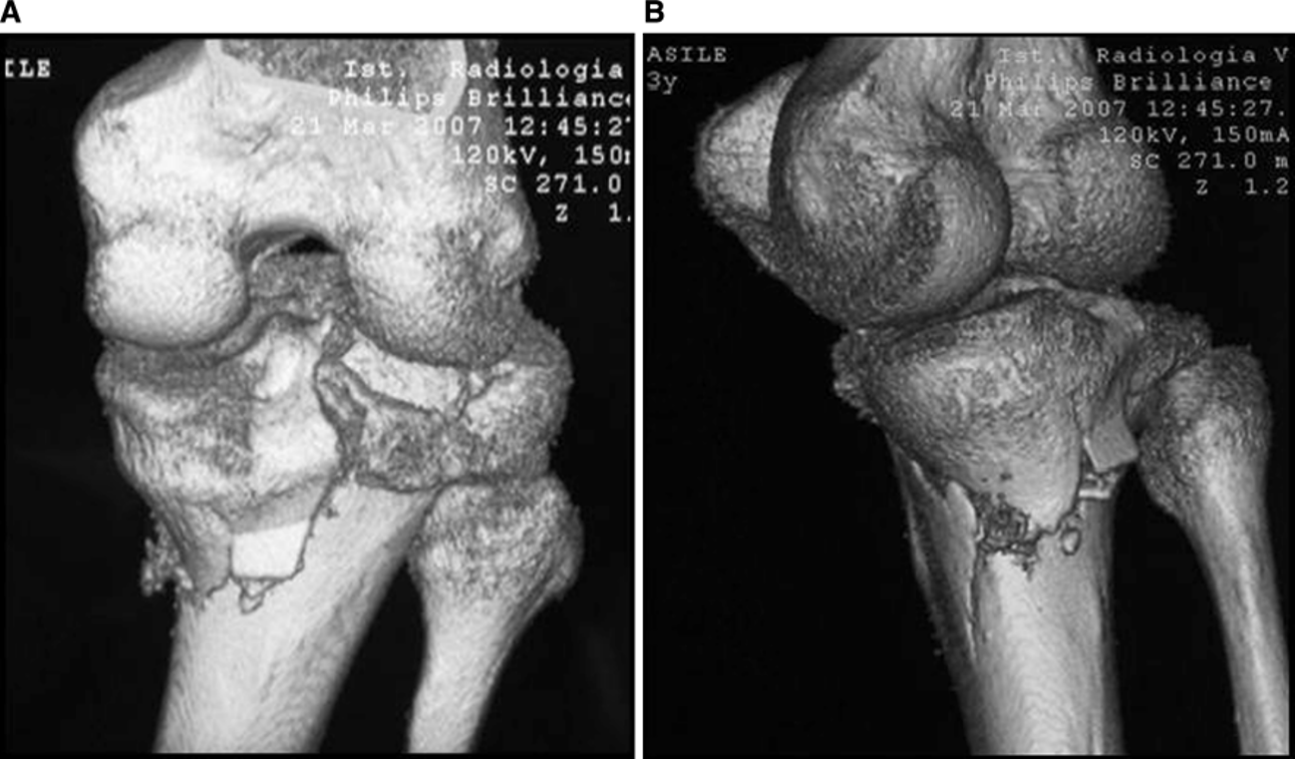
**a** Schatzker type IV fracture, CT 3D reconstruction. **b** Schatzker type IV fracture, CT 3D reconstruction

**Fig. 5:**
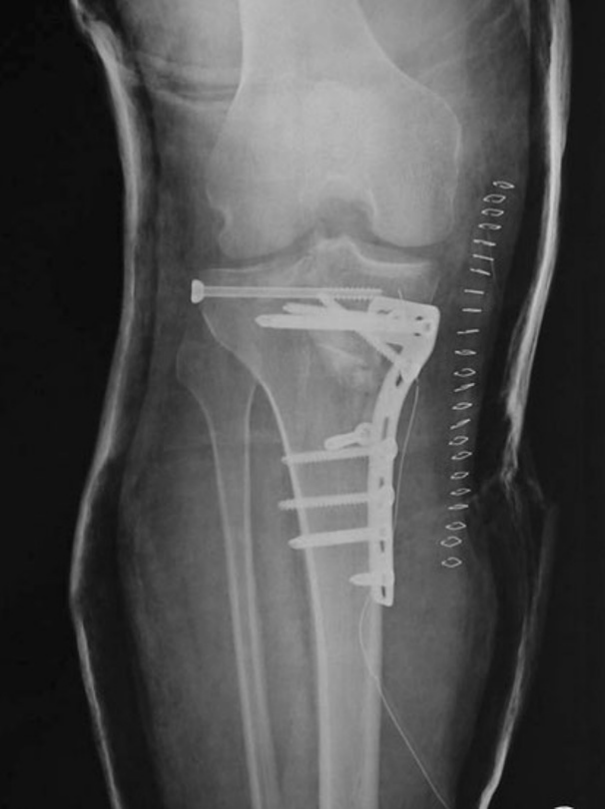
Schatzker type IV fracture, post-op X-rays (AP)

**Fig. 6:**
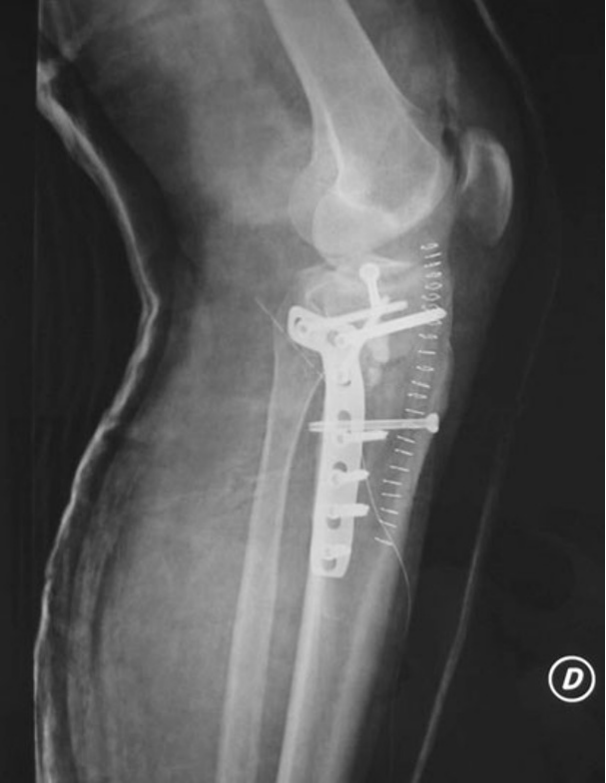
Schatzker type IV fracture, post-op X-rays (LL)

Schatzker IV type fractures (Figs. 1, 2, 3a–d, 4a, b, 5, 6) treated by ARIF technique demonstrated better results than those treated by ORIF. This may have been achieved because the procedure of avoided arthrotomy, with a temporary reduction by manipulation and confirmation of reduction by arthroscopy allowing surgical objectives to be accomplished with minimal damage to the capsule of the joint. On restoration of articular congruity, reduction was maintained with cannulated screws or, when needed, a medial plate applied without arthrotomy [38, 39]. This type of fracture is characterized by many associated injures (5 medial meniscus tears, 3 ACL, 2 PCL and 1 LCL ruptures). In ARIF group, the meniscal lesions were treated after the reduction and fixation of the fracture, whereas the ACL was reconstructed after fracture union. The LCL lesion occurred in ORIF group which was treated at the same time. There were late complications observed: plate intolerance due to interference at the insertion of the hamstrings tendons were treated by removal.

**Fig. 7:**
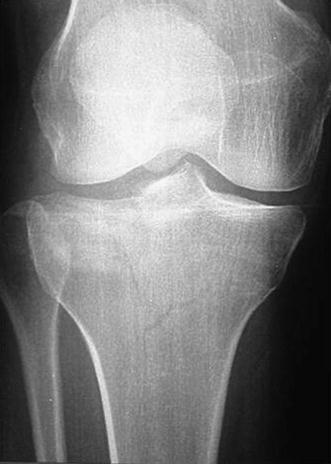
Schatzker type V fracture, pre-op X-rays (AP)

**Fig. 8:**
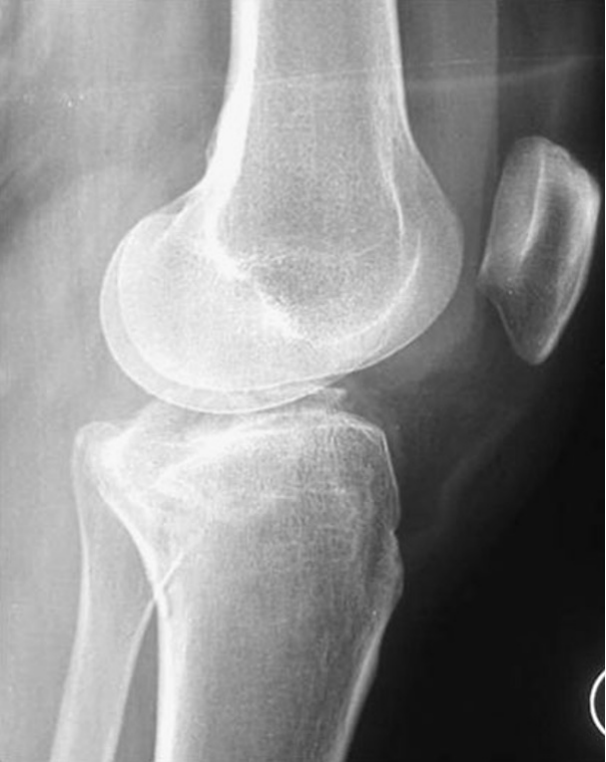
Schatzker type V fracture, pre-op X-rays (LL)

**Fig. 9:**
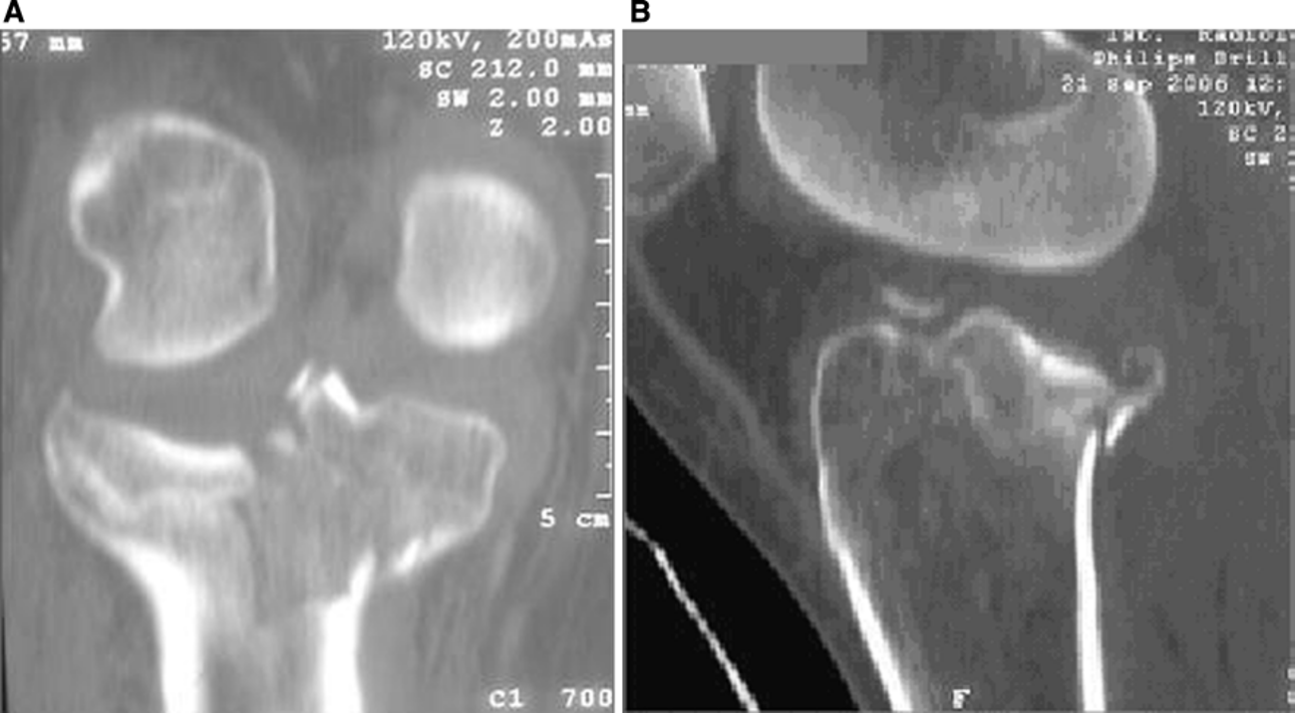
**a** Schatzker type V fracture, CT scan. **b** Schatzker type V fracture, CT scan

**Fig. 10:**
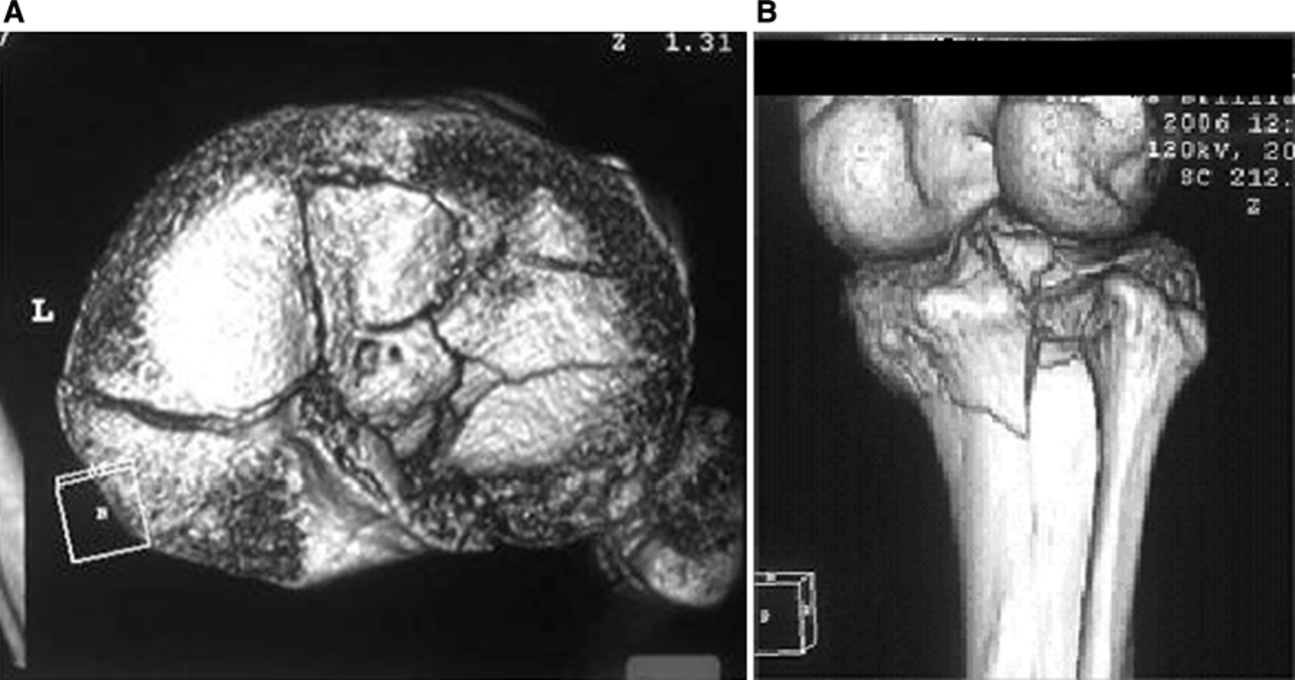
**a** Schatzker type V fracture, CT 3D reconstruction. **b** Schatzker type V fracture, CT 3D reconstruction

**Fig. 11:**
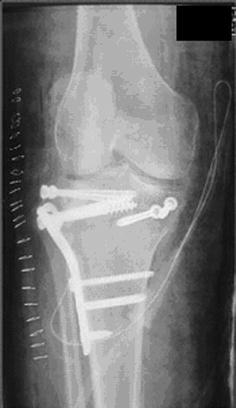
Schatzker type V fracture, post-op X-rays (AP)

**Fig. 12:**
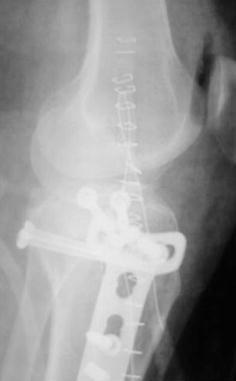
Schatzker type V fracture, post-op X-rays (LL)

**Fig. 13:**
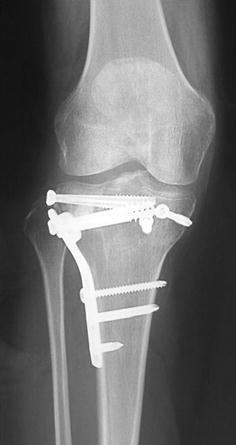
Schatzker type V fracture, 4 months X-rays (AP)

**Fig. 14:**
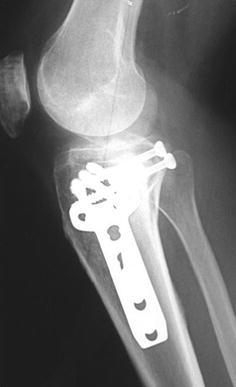
Schatzker type V fracture, 4 months X-rays (LL)

**Fig. 15:**
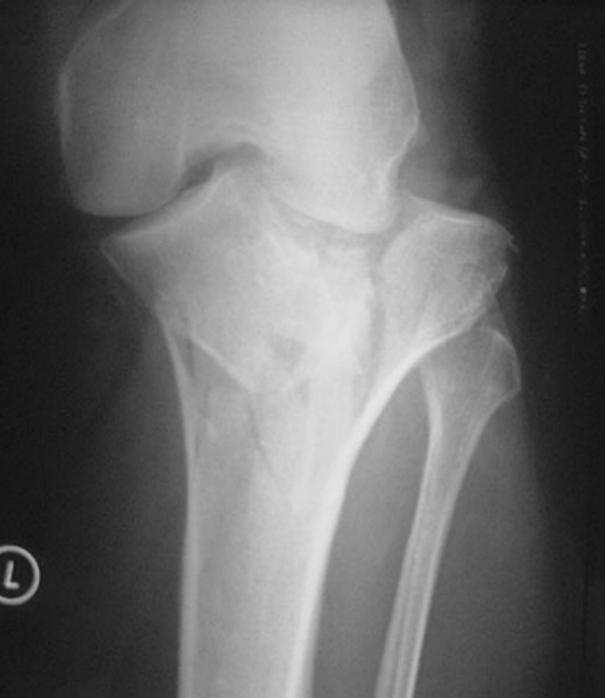
Schatzker type VI fracture, pre-op X-rays (AP)

**Fig. 16:**
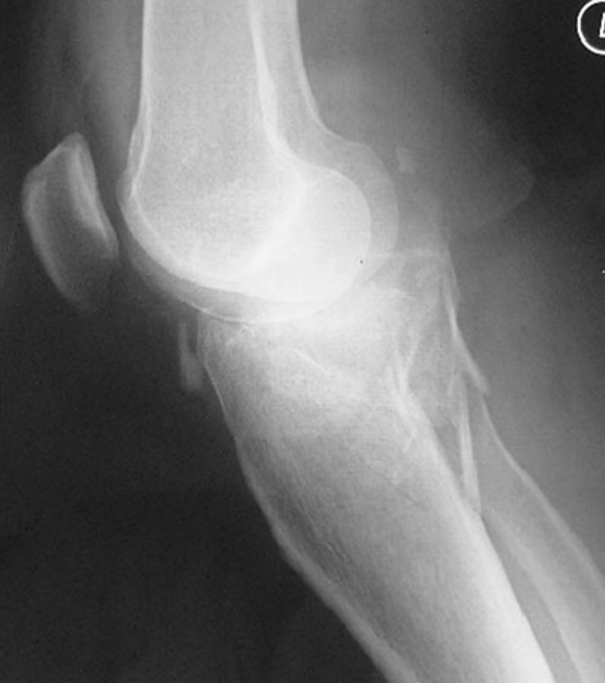
Schatzker type VI fracture, pre-op X-rays (LL)

**Fig. 17:**
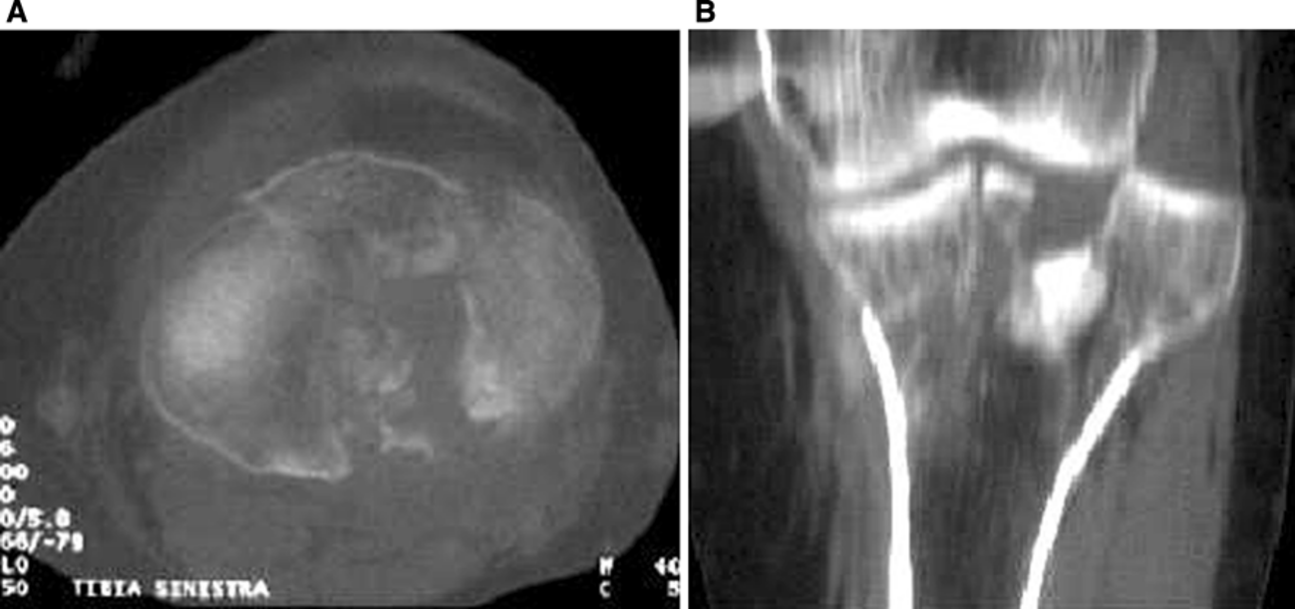
**a** Schatzker type VI fracture, CT scan. **b** Schatzker type VI fracture, CT scan

**Fig. 18:**
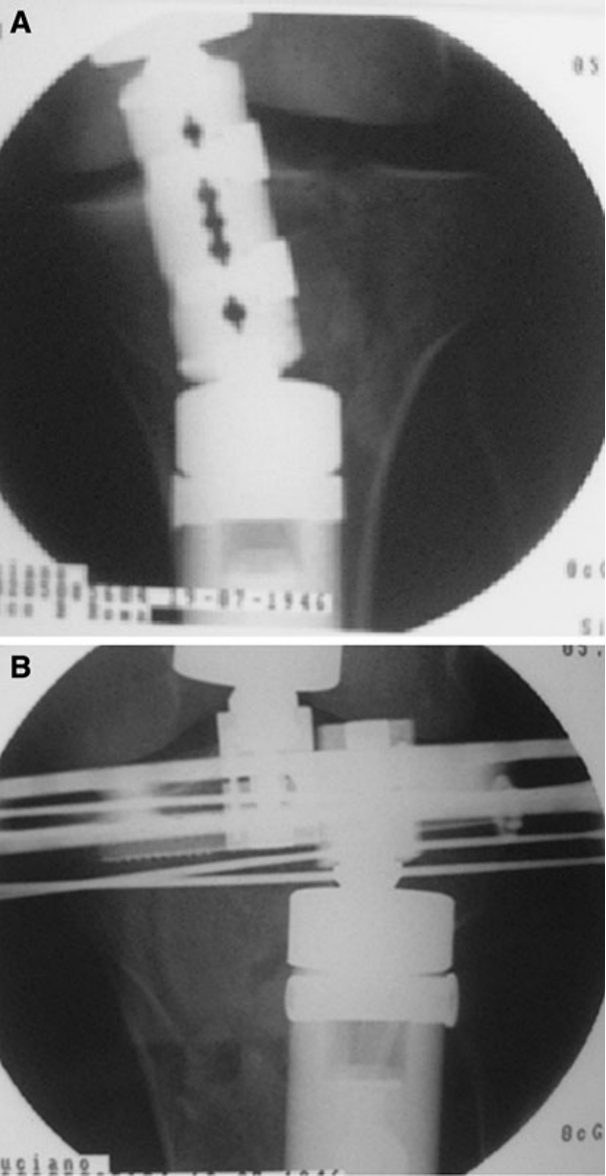
**a** Schatzker type VI fracture, temporary spanning fixator. **b** Schatzker type VI fracture, temporary spanning fixator

**Fig. 19:**
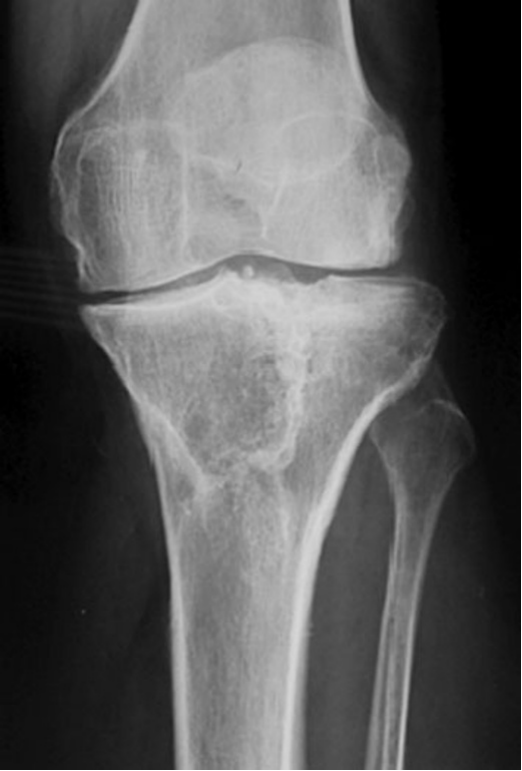
Schatzker type VI fracture, 1 year after X-rays (AP)

**Fig. 20:**
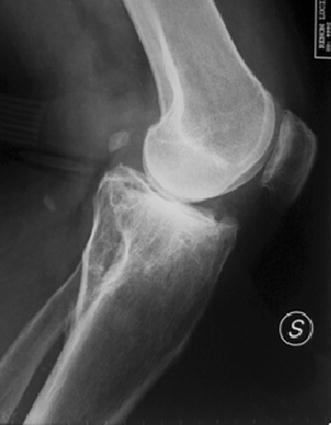
Schatzker type VI fracture, 1 year after X-rays (LL)

Cassard et al. described 26 patients with Schatzker types I–IV fractures treated arthroscopically and concluded that the results were as good as or better than from ORIF [40]. In this series, ARIF treatment of Schatzker V (Figs. 7, 8, 9a, b, 10a, b, 11, 12, 13, 14) and VI type fractures (Figs. 15, 16, 17a, b, 18a, b, 19, 20) was carried out in selected cases, typified by a lower degree of comminution, because the water pressure from joint distension could lead to loss of loose cartilage fragments. There was an advantage in performing a single lateral access, arthroscopically inspecting the cartilage, cruciate ligaments and menisci. An image intensifier was used to check the reduction previously obtained using K wires or cannulated screws. A lateral plate was finally applied with avoidance of arthrotomy and direct open access.

Two cases of deep vein thrombosis were observed in Schatzker VI fractures, 1 in group A and 1 in group B, which resolved after appropriate therapy with low-molecular weight heparin. There were neither superficial nor deep infections in patients treated by ARIF technique. Two patients of the ORIF group with Schatzker V type fractures had superficial infections and wound problems which resolved completely with appropriate antibiotic therapy. There were 2 deep infections also in ORIF group. This complication is well noted by several authors of reports of these complex fractures. Stamer et al. reported a review of 23 knees in 22 patients with Schatzker type VI injuries with a 100 % infection rate when extensive dissection was performed to allow the use of a plate in conjunction with external fixation. Six of the 23 knees (26 %) had complications, including 3 deep wound infections, 1 deep vein thrombosis, 1 malunion and 1 pin tract infection [41]. Barei et al. has reported an 8.4 % deep infection rate; the Canadian Orthopaedic Trauma Society reported 17 % [17, 42]. This review has confirmed a major rate of complications seen following open reduction and internal fixation of these difficult fractures, despite the use of ARIF technique. Deep infection in the ORIF group followed 14 % of the types V and VI fractures. However, no infection was observed in the same type of fractures in the ARIF group but it is acknowledged that these were of the lower comminution types.

There were frequent diagnoses of associated lesions: 11 meniscal tears, 3 ACL, 2 MCL and 1 PCL lesions in Schatzker V type of fractures; 7 meniscal tears, 4 ACL, 4 MCL lesions in Schatzker type VI injuries. The meniscal lesions in ARIF group were treated at the same time as the fracture, while the same lesions in ORIF group were treated at a subsequent surgery. For all cases, ruptures of the ACL were treated after fracture healing. The MCL was not repaired in any patient. The single PCL lesion was left untreated because it was not clinically relevant.

The incidence of associated injures with tibial plateau fractures in this series is similar to that reported in the literature [38, 43]. The Canadian Orthopaedic Trauma Society suggested that diagnosis of associated soft-tissue lesions and their subsequent treatment improved clinical outcome [2, 17, 44]. Chan et al. and Hung et al., evaluating soft-tissue lesions, claimed that these are minor injuries but could compromise the final results. They found that their concomitant treatment, during fractures reduction and fixation, may lead to difficulties. This encompassed both meniscal and other soft-tissue lesions including intra-articular ligaments. We hold the view that treatment of meniscal lesions at the same time as fracture reduction could improve the surgical and clinical outcome, whilst ligamentous reconstruction (ACL and PCL) may be technically difficult owing to the fracture comminution and the presence of internal fixation. For these reasons, the surgeon may incorrectly position and fix the new ligament. Another problem would be the contrasting rehabilitation regimes for fracture or ligament reconstruction [45, 46].

Post-traumatic arthritis is a common sequela of tibial plateau fractures [38, 47, 48]. Fifteen (68 %) patients with Schatzker V and VI types of fracture were affected by joint degeneration due to malalignment, non-union and severe cartilage damage. Four were treated with knee replacements [49–51].

## Conclusions

There were no differences between ARIF and ORIF treatment for type I tibial plateau fractures. We found ARIF treatment preferable when meniscal tears were present as it gave opportunity for simultaneous treatment. In cases of Schatzker II, III and IV fractures, there was a small difference in clinical outcomes in favour of ARIF but not statistically significant. In Schatzker V or VI fractures, ARIF treatment was limited to less comminuted fractures and showed less incidence of infection. Mid-to-long-term clinical results are influenced by the development of post-traumatic arthritis and this itself was related to the severity of the initial cartilage damage, subsequent malalignment and non-union.
